# Improving the performance of lexicon-based review sentiment analysis method by reducing additional introduced sentiment bias

**DOI:** 10.1371/journal.pone.0202523

**Published:** 2018-08-24

**Authors:** Hongyu Han, Yongshi Zhang, Jianpei Zhang, Jing Yang, Xiaomei Zou

**Affiliations:** College of Computer Science and Technology, Harbin Engineering University, Harbin, Heilongjiang Province, China; Tampere University of Technology, FINLAND

## Abstract

Sentiment analysis is widely studied to extract opinions from user generated content (UGC), and various methods have been proposed in recent literature. However, these methods are likely to introduce sentiment bias, and the classification results tend to be positive or negative, especially for the lexicon-based sentiment classification methods. The existence of sentiment bias leads to poor performance of sentiment analysis. To deal with this problem, we propose a novel sentiment bias processing strategy which can be applied to the lexicon-based sentiment analysis method. Weight and threshold parameters learned from a small training set are introduced into the lexicon-based sentiment scoring formula, and then the formula is used to classify the reviews. In this paper, a completed sentiment classification framework is proposed. SentiWordNet (SWN) is used as the experimental sentiment lexicon, and review data of four products collected from Amazon are used as the experimental datasets. Experimental results show that the bias processing strategy reduces polarity bias rate (PBR) and improves performance of the lexicon-based sentiment analysis method.

## Introduction

Social media sites have been growing exponentially in recent years. They have already become important and popular platforms for people to express their emotions, opinions, experiences etc. There are numerous UGC published on social media every day. Such UGC comes into being a significant source to dig out useful information such as opinions of customers to a specific product or service, comments of the public about the existing or newly proposed policies, and voting tendencies of the electors. The opinions in the UGC are increasing used to make decisions by individuals, organizations and government agencies. It is also much easier to get individual feedbacks timely from the UGC instead of conducting a questionnaire.

Sentiment analysis is the computational study of people’s opinions, sentiments, emotions, and attitudes, is also known as opinion mining [1]. Many sentiment analysis techniques have been proposed in last few years. Polarity detection is the most common form of sentiment analysis. It can be classified into two categories: supervised learning approaches and lexicon-based approaches [2]. However, the study [3] indicates that many methods present more positive values than negative values, especially the lexicon-based method. Sentiment bias widely exists in lexicon-based methods especially when general purpose sentiment lexicons are used, which leads to poor and imbalanced polarity classification results.

In this paper, we propose a novel method to process sentiment bias, and a lexicon-based sentiment analysis framework is designed with the sentiment bias processing strategy. Weight and threshold parameters are introduced to re-evaluate sentiment score of the reviews. The weight parameter ensures that the positive and negative expressions are weighted properly and the threshold parameter makes sure that the PBR of the classification results is reduced. Small-scale training set is used to learn optimal parameters. The proposed method can significantly improve the performance of lexicon-based sentiment analysis method by reducing sentiment bias.

The main contributions of this paper are summarized as follows:

1. We present a sentiment bias processing strategy for the lexicon-based sentiment analysis method.
2. We design a SWN-based sentiment analysis framework which uses the proposed sentiment bias processing strategy.
3. We conduct experiments on four Amazon product review datasets, and results demonstrate the feasibility and effectiveness of the proposed method.

The rest of the paper is organized as follows. Section 2 discusses the related work. In Section 3, the proposed approach is introduced in detail. Section 4 presents and analysis the experiments and results. Finally, we conclude the paper in Section 5.

## Related work

### Supervised learning method

Machine learning is the most common method used in the supervised learning method for sentiment analysis. Many machine learning algorithms such as Naive Bayes, Maximum Entropy Classification, Support Vector Machines, Artificial Neural Network, Decision Tree, K-Nearest Neighbor, and Ensemble Learning are naturally used, since the sentiment polarity detection can be seen as a classification task.

The study [4] makes use of movie reviews as the experimental dataset, and three machine learning methods (Naive Bayes, maximum entropy classification, and support vector machines) are employed to tackle the sentiment classification problem. The study [5] proposes an ANN-based method for document-level sentiment classification. The study [6] conducts a comparative assessment of the performance of three popular ensemble methods (Bagging, Boosting, and Random Subspace) based on five base learners (Naive Bayes, Maximum Entropy, Decision Tree, K Nearest Neighbor, and Support Vector Machine) for sentiment classification. Experimental results show that ensemble methods can get better results than base learners [7, 8]. However, sufficient labeled training data is required in the supervised learning methods for sentiment analysis, and the training data acquisition becomes a very laborious process [9].

### Lexicon-based method

Sentiment lexicon is collection of words (or phrases) that convey feelings [1]. Each entry in the sentiment lexicon is associated with its sentiment orientation and strength [10]. Entries in the lexicon can be divided into three categories according to their sentiment orientations, such as positive, negative, and neutral. There are several well-known general purpose constructed sentiment lexicon such as SWN [11], Multi-Perspective Question Answering (MPQA) [12], General Inquirer (GI) [13], and Opinion lexicon (OL) [14].

The lexicon-based approach calculates the final sentiment tendency value of a review by rating the sentiment tendency of each word or phrase in a given review [2]. Numerous studies are presented, which mainly study how to assign each sentiment expression. In the study [14], positive words are assigned +1, and negative words are assigned -1, negation words reverse the sentiment value. The study [15] makes more fine-grain assignment, sentiment expressions are assigned from -5 to +5 (0 is not used), intensifiers and diminishers are handled. The lexicon-based approach is more appropriate if lack of sufficient tagged data.

### Sentiment bias

Sentiment bias is widely existing in the current sentiment analysis methods [3]. Although sentiment bias has been identified, little work has studied at this point. The study [16] proposes a bias-aware thresholding(BAT) sentiment analysis method, in which PBR is reduced by utilizing threshold parameter to determine whether a review is positive or negative (If the sentiment score of a review is larger than the threshold value, the review is classified as positive, otherwise, it is classified as negative). The threshold vlaue is learned from a small training set. While the influence of the weights of positive and negative expressions on the predicted result is not taken into account. In the study [15], Semantic Orientation CALculator (SO-CAL) is proposed, it uses dictionaries of words annotated with their semantic orientation (polarity and strength), incorporates intensification and negation, and the final sentiment orientation value of any negative expression by 50%, this method can improve the lexicon-based sentiment classification performance in some domains, but the improvement is limited because fixed weight parameter can not be proper for all domains, and PBR of the classification result has not been effectively reduced.

## Methodology

**Fig 1:**
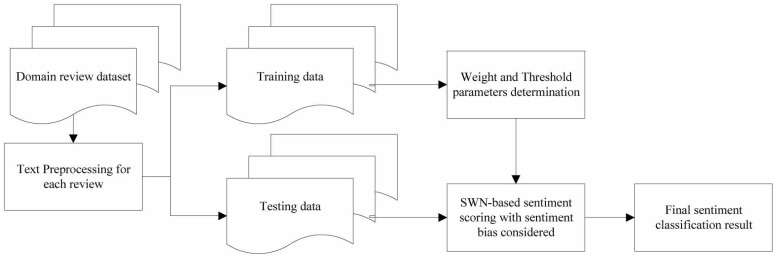
Overview of the proposed sentiment classification framework.

An overview of the proposed sentiment classification framework is presented in Fig 1. The framework initiates by acquiring domain specific review datasets from online data repositories (datasets we used in this paper will be introduced in Section 4). Then text preprocessing is conducted for all the reviews in the dataset. In the next phase, training set (reviews with sentiment polarity labels) is used to learn optimal parameters. Finally, we get the sentiment rating formula (weight and threshold parameters are assigned), which is used to perform the sentiment polarity classification.

### Text preprocessing

Text preprocessing is necessary to the proposed sentiment classification framework. Three steps are used to process the reviews in the dataset.

#### Remove URLs and specific symbols

URLs in reviews convey no sentiment information but disturb the sentiment analysis, so we remove them. For example, word “good” in “http://baidu.com/?aldtype=85#en/zh/good” will obviously affect the sentiment rating result if it is considered, while it usually does not convey the reviewer’s opinion. Specific symbols (such as () @ # $ %, etc.) are widely existing in the reviews doing nothing but affect the word taken process, therefore we replace them with spaces.

#### Part of speech (POS)

POS Tagger is used to assign part of speech to each word in the text (and other tokens), such as noun, verb, adjective, etc. [17]. A word may have variety sentiment values according to its POS, as the same word may play different roles according to its POS. For example, “good” is mostly convey positive feelings when it is used as an adjective but no positive or negative feelings when it is used as a noun [10]. Since the POS Tagger makes use of Penn POS Tag [18] to tag the word in the text and SWN has only four POS tags such as a, r, v, and n, we just consider the word with the four POS tags, and we need to turn tags of the words from Penn POS Tags to SWN POS Tags, corresponding relations of Penn POS Tags and SWN POS Tags are displayed in Table 1.

**Table 1:** Comparison table of POS tags.

Penn POS	Description	SWN POS
JJ	Adjective	a
JJR	Adjective, comparative	a
JJS	Adjective, superlative	a
RB	Adverb	r
RBR	Adverb, comparative	r
RBS	Adverb, superlative	r
VB	Verb, base form	v
VBD	Verb, past tense	v
VBG	Verb, gerund or present participle	v
VBN	Verb, past participle	v
VBP	Verb, non-3rd person singular present	v
VBZ	Verb, 3rd person singular present	v
NN	Noun, singular or mass	n
NNS	Noun, plural	n
NNP	Proper noun, singular	n
NNPS	Proper noun, plural	n

#### Lemmatization

Lemmatization is used to map word-forms to a standardized lexicon entry [19]. Thus it is an indispensable procedure to address the word mismatch problem. We use the SWN as the sentiment lexicon here, and we utilize the NLTK (Natural Language Toolkit) [20] to conduct the Lemmatization procedure. It will obviously improve the quality of the sentiment rating results.

### SWN-based sentiment scoring process

SentiWordNet is a well-known general purpose sentiment lexicon. The latest version is SentiWordNet 3.0. Table 2 shows part of SentiWordNet 3.0.

**Table 2:** Part of SentiWordNet 3.0.

POS	ID	PosScore	NegScore	SynsetTerms	Gloss
a	1936528	0	0.75	notional#1 imaginary#1 fanciful#2	not based on fact; unreal; “the falsehood about some fanciful secret treaties”- F.D.Roosevelt; “a small child’s imaginary friends”; “to create a notional world for oneself”
a	1796304	0.5	0.25	fanciful#3	having a curiously intricate quality; “a fanciful pattern with intertwined vines and flowers”
a	643598	0.5	0	notional#3 fanciful#1	indulging in or influenced by fancy; “a fanciful mind”; “all the notional vagaries of childhood”
n	4268142	0	0	sparker#1 spark_arrester#2	a wire net to stop sparks from an open fireplace or smokestack
r	449609	0.25	0	sentimentally#1	in a sentimental manner; “‘I miss the good old days,’ she added sentimentally”
v	2771169	0.125	0	light_up#3 clear_up#4 clear#3 brighten#2	become clear; “The sky cleared after the storm”

The WordNet [21] synsets are uniquely identified by (POS, ID) pairs. *PosScore* and *NegScore* are the positivity and negativity of the corresponding synset. SynsetTerms column shows the included terms with the sense numbers. The sentiment score of the synset can be calculated by the following expression:

$$\begin{array}{c} SynsetScore=PosScore-NegScore \end{array}$$ (1)

For a term with the specific POS tag, if *n* synsets contain it, sentiment score of the term can be calculated by the following expression:

$$\begin{array}{c} TermScore=\frac{\sum_{r=1}^{n}SynsetScore(r)/r}{\sum_{r=1}^{n}1/r} \end{array}$$ (2)

where *r* is the sense number. The term with the specific POS can be recognized as positive, negative or neutral depending on whether its *TermScore* is greater than, less than or equal to zero, respectively.

For example, all the SynsetTerms containing “fanciful” with POS “a” are listed in Table 2. TermScore of “fanciful#a” is calculated as follow:

$$\begin{array}{c} TermScore\left(fanciful\#a\right)=\frac{(0.5-0)/1+(0-0.75)/2+(0.5-0.25)/3}{1/1+1/2+1/3}=0.1136 \end{array}$$

(0.5-0) is *SynsetScore* of the synset which contains “fanciful” with sense number 1, and corresponding POS of the synset is “a”. “fanciful#a” is positive as the TermScore of “fanciful#a” is greater than zero.

After the preprocessing phrase, the sentiment score for each term in the review is assigned according to Eqs (1) and (2). If a term is not in the SWN, we assume that its sentiment score is 0. If a negation word appears in front of a term, we reverse the sentiment value of the term. The sentiment score of the target review is obtained via adding up all term sentiment scores, as it is shown in the following formations:

$$\begin{array}{c} PosScore\left(r\right)=\sum_{i=1}^{m}TermScore\left(T_{i}\right) \end{array}$$ (3)

$$\begin{array}{c} NegScore\left(r\right)=\sum_{j=1}^{n}TermScore\left(T_{j}\right) \end{array}$$ (4)

$$\begin{array}{c} SentiScore(r)=PosScore(r)+NegScore(r) \end{array}$$ (5)

where *r* is a review which contains *m* positive terms and *n* negative terms, *PosScore*(*r*) and *NegScore*(*r*) represent the positivity and negativity of the corresponding review *r*, and *SentiScore*(*r*) represents the final sentiment score of the review *r*.

### Sentiment bias processing strategy

To process the sentiment bias, we introduce weight and threshold parameters to the sentiment rating formation for review sentiment analysis as follow:

$$\begin{array}{c} SentiScore\prime \left(r\right)=\alpha \times PosScore\left(r\right)+\left(1-\alpha \right)\times NegScore\left(r\right)+t \end{array}$$ (6)

where *SentiScore*′(*r*) is the sentiment score of review *r*, weight parameter *α* range from 0 to 1. *t* is a threshold parameter. Then sentiment score of each review can be assigned according to Eq (6). If the sentiment score is larger than zero, the corresponding review is classified as positive, otherwise, it is classified as negative.

A small training dataset is used to determine the optimal parameters (weight *α* and threshold *t*). The values of weight *α* and threshold t which make the lexicon-based sentiment analysis method get the highest F-measure and lowest PBR are record as the optimal values of them. PBR can be defined according to reference [16] as Eq (7), and F-measure is presented as Eq (10).

$$\begin{array}{c} PBR=|\frac{FP-FN}{TP+FN+TN+FP}| \end{array}$$ (7)

$$\begin{array}{c} Sensitivity=\frac{TP}{TP+FN} \end{array}$$ (8)

$$\begin{array}{c} Specificity=\frac{TN}{TN+FP} \end{array}$$ (9)

$$\begin{array}{c} F-measure=\frac{2\times Sensitivity\times Specificity}{Sensitivity+Specificity} \end{array}$$ (10)

where *TP*, *FP*, *TN*, and *FN* correspond to the number of truly identified positive reviews, wrongly identified positive reviews, truly identified negative reviews and wrongly identified negative reviews, respectively.

Alforithm 1 demonstrates how to learn optimal parameters from the training set. For a specific *α*_j_, first we get the weighted sentiment scores of the reviews in the training set T, then the corresponding optimal threshold *t*_j_ is acquired with the help of Algorithm 2 which is an sentiment polarity classification algorithm with a specific threshold *t*. Finally, we could get optimal weight *α* and threshold *t* by incremental assignment of *α* at which F-measure gets the highest value.

Algorithm 2 implements sentiment polarity classification by utilizing Eq (6). If the sentiment score is larger than zero, the corresponding review is classified as positive, otherwise, it is classified as negative. In this paper, we make use of balanced training set in which the number of positive and negative reviews are equal. So we can get the optimal threshold *t* at which *PBR* = 0, it can be found accurately and efficiently by using bisection method.

**Algorithm 1** Parameter Assignments

**Input**: Training set *T* = {*r*_1_, *r*_2_, *r*_3_ … *r*_n_}

**Output**: *α*, *t*

1: **for** *α*_j_ in range (0,1) **do**

2:  Initialize Set *S* = {*s*_k_|*s*_k_ = 0, 1 ≤ *k* ≤ *n*}

3:  **for** *r*_i_ in *T* **do**

4:   *s*_i_ ← *α* × *PosScore*(*r*_i_) + (1 − *α*) × *NegScore*(*r*_i_)

5:  **end for**

6:  Seek the *t*_j_ at which *PBR* = 0 according to Algorithm 2

7:  Record *α*_j_, *t*_j_, *F* − *measure*(*T*, *α*_j_, *t*_j_)

8: **end for**

9: Seek the (*α*_j_, *t*_j_) at which *F* − *measure*(*T*, *α*_j_, *t*_j_) is highest

10: **return** (*α*, *t*) which get in step 9

**Algorithm 2** Sentiment Polarity Determination with a Specific Threshold *t*

**Input**: Score set *S* (*s*_i_ is the sentiment score of corresponding review *r*_i_ from Training Set *T*, it is weighted with a specific weight *α* and assigned in Step 3-5 of Algorithm 1.); threshold *t*

**Output**: Label Pos or Neg for *r* in *T*

1: **for** *s*_i_ in *S* **do**

2:  **if** *s*_i_ + *t* > 0 **then**

3:   *Polarity*(*r*_i_) ← *Pos*

4:  **else**

5:   *Polarity*(*r*_i_) ← *Neg*

6:  **end if**

7: **end for**

## Experimental evaluation

In this section, first we introduce the experimental dataset, then evaluate the performance of the proposed method. Additionally, the influence of training data size on the experimental results is analyzed.

### Experimental dataset

The dataset we used is Amazon product review dataset, it can be found at https://www.cs.jhu.edu/~mdredze/datasets/sentiment/. It is a benchmark dataset which is widely used in sentiment analysis. Review datasets for several products are included, which are collected from Amazon.com. There are 1000 positive and 1000 negative tagged reviews in each product review dataset [22]. Four product (Books, DVD, Electronics, and Kitchen) review datasets are used in the experiments.

### Performance evaluation

To evaluate the proposed sentiment analysis framework, we compare it against BAT and SO-CAL. The corresponding weight parameter values of BAT and SO-CAL are 0.5 and 0.4, respectively. Evaluation measures used in this paper are PBR, F-measure, and Accuracy which are presented in Eqs (7), (10) and (11).

$$\begin{array}{c} Accuracy=\frac{TP+TN}{TP+FP+TN+FN} \end{array}$$ (11)

To each experimental dataset, 200 positive and 200 negative reviews (20% of the dataset) are randomly selected as the training set. The optimal weight *α* and threshold *t* are assigned by utilizing Alforithm 1 and Alforithm 2. Then sentiment score of each review can be assigned according to Eq (6). If the sentiment score is larger than zero, the corresponding review is classified as positive, otherwise, it is classified as negative. The performance of BAT, SO-CAL and the proposed methods are measured, the experiments are repeated five times (training dataset and testing dataset are randomly selected) and the average results are given in Table 3.

**Table 3:** Performance evaluation on four datasets.

	BAT			SO-CAL			Proposed		
	Accuracy	F-measure	PBR	Accuracy	F-measure	PBR	Accuracy	F-measure	PBR
DVD	64.74%	64.78%	0.28%	67.41%	68.00%	6.40%	**69.79%**	**69.88%**	**0.02%**
Electronics	65.58%	65.69%	1.99%	66.95%	67.20%	4.10%	**68.72%**	**68.80%**	**1.68%**
Books	62.74%	62.82%	1.36%	65.98%	66.90%	7.90%	**68.17%**	**68.26%**	**0.70%**
Kitchen	69.62%	69.67%	**0.05%**	67.81%	68.45%	6.65%	**71.41%**	**71.44%**	0.76%

The following observations and conclusions can be made in Table 3:

1. The proposed sentiment analysis framework performs well than both BAT and SO-CAL methods in Accuracy and F-measure on all the datasets.
2. The SO-CAL method performs well than BAT on Books, DVD, and Electronics datasets but worse on Kitchen dataset. We can see that fixed weight may make the lexicon-based sentiment analysis method gets well performance in some domains but it could not be suitable for all domains.
3. The proposed method and BAT get smaller PBR values than SO-CAL. The threshold parameter could reduce PBR of the lexicon-based sentiment framework.

We analyse the results as follows:

1. The threshold parameter *t* makes the proposed method get same ability with BAT in reducing PBR.
2. If 0.5 is the optimal value of weight *α*, the proposed method get the same result with BAT. If 0.4 is the optimal value of weight *α*, the proposed method can achieve better results than SO-CAL by using the threshold parameter *t*. Therefore, when optimal weight and threshold values are assigned, the proposed method could get equality or superior performance to BAT and SO-CAL.

To sum up, the method proposed in this paper is superior to BAT and SO-CAL methods in F-measure and Accuracy, and it gets the same ability with BAT in reducing PBR.

### Parameter analysis

**Fig 2:**
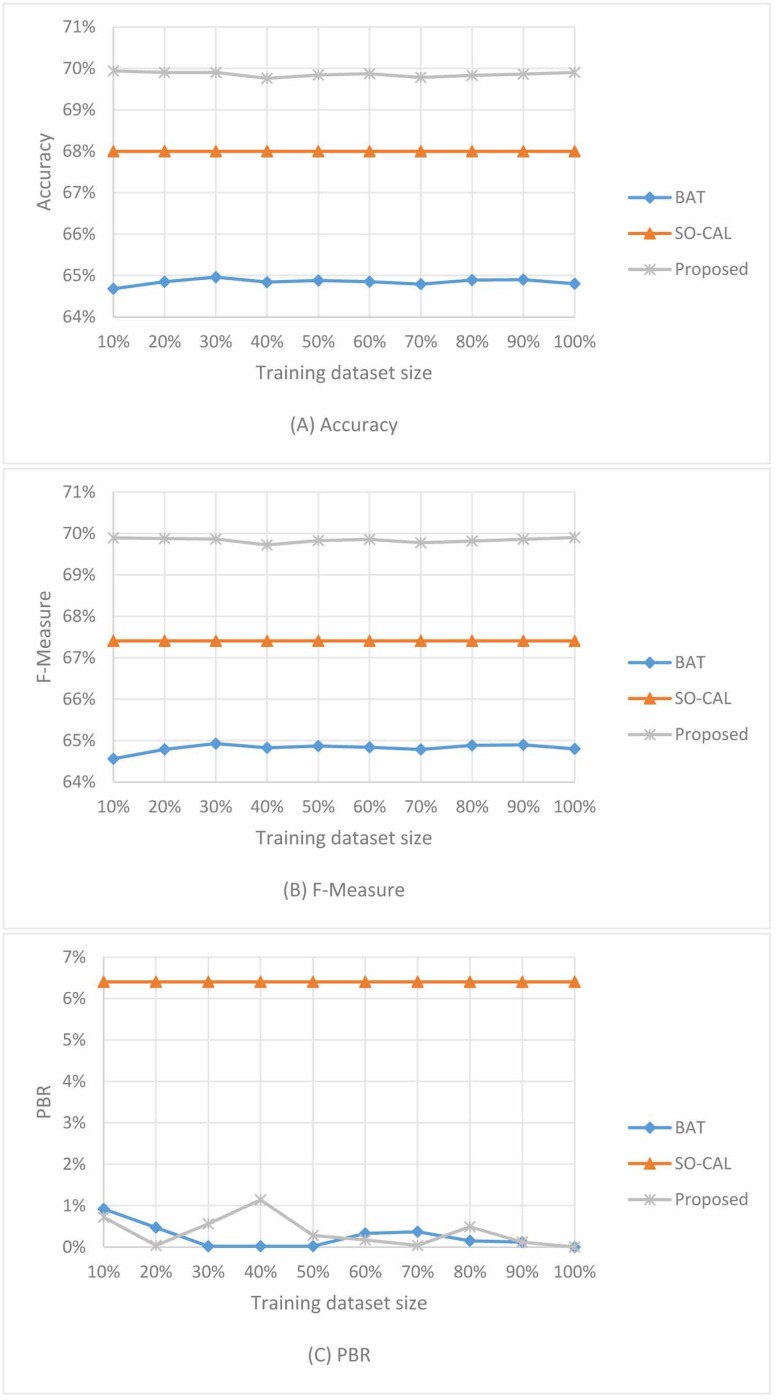
The influence of training set size on experimental results. (A): Accuracy. (B): F-measure. (C): PBR.

In this section, experiments are conducted to analyse the influence of the amount of the selected positive and negative reviews used for training weight and threshold parameters. We select the DVD dataset as the experimental dataset here. 50% of the reviews (500 positive and 500 negative reviews) in the DVD dataset are randomly selected as training set and the remaining 50% reviews are used as the testing set. Then variety percentages reviews from the training set are used to learn weight and threshold parameters. The influence of the training dataset size on the performances of the proposed sentiment classification framework, BAT and SO-CAL methods are presented in the Fig 2.

The following observations can be made in Fig 2:

1. Both BAT and the proposed sentiment classification framework get stability performance in Accuracy, F-measure and PBR along with the training set size changes.
2. Even when a small scale training set (50 positive and 50 negative reviews) is used, the proposed method could perform well.
3. Along with the training set size changes, the proposed sentiment classification framework outperforms BAT and SO-CAL in Accuracy and F-measure, and get the same degree of PBR with BAT.

SO-CAL makes use of fixed weight parameter, no additional training set is needed, so its performance on testing set is invariable. Small-scale tagged training set from the specific domain dataset has the same sentiment bias characteristics with the entire dataset, it makes the proposed sentiment analysis framework could get stability performance. The similar threshold parameter training strategy makes the BAT and the proposed approach have equality ability in reducing PBR. As the proposed approach could get stability performance along with the training dataset size changes, thus we can choose a proper value of the training set size easily.

## Conclusion

Sentiment bias in review sentiment analysis significantly affects the sentiment polarity detection, which leads to reviews being wrongly classified. Recent literature has shown that sentiment bias widely exists in a variety of sentiment analysis methods, especially the lexicon-based sentiment analysis methods which make use of general purpose sentiment lexicons. However, few works have been addressing this problem.

In this paper, we propose a sentiment bias processing strategy for lexicon-based sentiment analysis, and we construct a lexicon-based sentiment analysis framework with the proposed sentiment bias processing strategy. Specifically, weight and threshold parameters are introduced into the proposed lexicon-based sentiment analysis method to process sentiment bias. Weight parameter makes sure that positivity and negativity of the reviews are weighted properly, threshold parameter balances the classification result and reduces the PBR. The optimal values of the parameters can be learned from a small training set accurately. Experiments are carried out on four review datasets which are randomly selected from Amazon product review dataset. The performance evaluation experiments show that the proposed sentiment analysis framework gets same ability with BAT and outperforms SO-CAL in reducing sentiment bias, and it outperforms BAT and SO-CAL in Accuracy and F-measure. Additionally, the influence of training dataset size on the performance of the proposed sentiment analysis framework is analyzed by conducting experiments on DVD review dataset, results show that the proposed sentiment analysis framework could get robust performance using training sets of varying sizes.
